# Nitrogen and sulfur cycling driven by Campylobacterota in the sediment–water interface of deep-sea cold seep: a case in the South China Sea

**DOI:** 10.1128/mbio.00117-23

**Published:** 2023-07-06

**Authors:** Qing-lei Sun, Ke Xu, Lei Cao, Zengfeng Du, Minxiao Wang, Li Sun

**Affiliations:** 1 College of Life Science, Qingdao Agricultural University, Qingdao, China; 2 CAS and Shandong Province Key Laboratory of Experimental Marine Biology, Center for Ocean Mega-Science, Institute of Oceanology, Chinese Academy of Sciences, Qingdao, China; 3 Laboratory for Marine Biology and Biotechnology, Laoshan Laboratory, Qingdao, China.; 4 Deep Sea Research Center, Institute of Oceanology, Chinese Academy of Sciences, Qingdao, China; 5 Key Laboratory of Marine Geology and Environment, Institute of Oceanology, Center for Ocean Mega-Science, Chinese Academy of Sciences, Qingdao, China; 6 CAS Key Laboratory of Marine Ecology and Environmental Science, Institute of Oceanology, Center for Ocean Mega-Science, Chinese Academy of Sciences, Qingdao, China; 7 College of Earth and Planetary Sciences, University of Chinese Academy of Sciences, Beijing, China; University of Tennessee at Knoxville, Knoxville, Tennessee, USA

**Keywords:** cold seep, *Sulfurimonas*, *Sulfurovum*, Campylobacterota, sulfur cycle, nitrogen cycle

## Abstract

Chemoautotrophs within Campylobacterota, especially *Sulfurovum* and *Sulfurimonas*, are abundant in the seawater–sediment interface of the Formosa cold seep in the South China Sea. However, the *in situ* activity and function of Campylobacterota are unknown. In this study, the geochemical role of Campylobacterota in the Formosa cold seep was investigated with multiple means. Two members of *Sulfurovum* and *Sulfurimonas* were isolated for the first time from deep-sea cold seep. These isolates are new chemoautotrophic species that can use molecular hydrogen as an energy source and CO_2_ as a sole carbon source. Comparative genomics identified an important hydrogen-oxidizing cluster in *Sulfurovum* and *Sulfurimonas*. Metatranscriptomic analysis detected high expression of hydrogen-oxidizing gene in the RS, suggesting that H_2_ was likely an energy source in the cold seep. Genomic analysis indicated that the *Sulfurovum* and *Sulfurimonas* isolates possess a truncated sulfur-oxidizing system, and metatranscriptomic analysis revealed that *Sulfurovum* and *Sulfurimonas* with this genotype were active in the surface of RS and likely contributed to thiosulfate production. Furthermore, geochemical and *in situ* analyses revealed sharply decreased nitrate concentration in the sediment–water interface due to microbial consumption. Consistently, the denitrification genes of *Sulfurimonas* and *Sulfurovum* were highly expressed, suggesting an important contribution of these bacteria to nitrogen cycling. Overall, this study demonstrated that Campylobacterota played a significant role in the cycling of nitrogen and sulfur in a deep-sea cold seep.

**IMPORTANCE:**

Chemoautotrophs within Campylobacterota, in particular *Sulfurovum* and *Sulfurimonas*, are ubiquitous in deep-sea cold seeps and hydrothermal vents. However, to date, no *Sulfurovum* or *Sulfurimonas* has been isolated from cold seeps, and the ecological roles of these bacteria in cold seeps remain to be investigated. In this study, we obtained two isolates of *Sulfurovum* and *Sulfurimonas* from Formosa cold seep, South China Sea. Comparative genomics, metatranscriptomics, geochemical analysis, and *in situ* experimental study indicated collectively that Campylobacterota played a significant part in nitrogen and sulfur cycling in cold seep and was the cause of thiosulfate accumulation and sharp reduction of nitrate level in the sediment–water interface. The findings of this study promoted our understanding of the *in situ* function and ecological role of deep-sea Campylobacterota.

## INTRODUCTION

Deep-sea cold seeps and hydrothermal vents are typical chemoautotrophic ecosystems, where autotrophic microbes are primary producers (1). Chemoautotrophs within Campylobacterota (previously known as Epsilonproteobacteria), especially *Sulfurovum* and *Sulfurimonas*, are ubiquitous in cold seeps and hydrothermal vents. These bacteria commonly inhabit redox boundaries, such as reduced sediments (RS), microbial mats, and chimney walls (2), which are deeply influenced by the seepage or vent. *Sulfurovum* spp. is also an episymbiont and can colonize the gills of shrimps (3). At the time of writing, five *Sulfurovum* species have been published, all of which were isolated from deep-sea hydrothermal systems (4 - 8). For *Sulfurimonas*, nine species have been published and six species have been proposed (https://lpsn.dsmz.de/genus/sulfurimonas). Five of these species, that is, *Sulfurimonas autotrophica* OK10^T^, *Sulfurimonas paralvinellae* GO25^T^, *Sulfurimonas indica* NW8N^T^, ‘*Sulfurimonas hydrogeniphila*’ NW10 and ‘*Sulfurimonas sediminis*’ S2-6 (9 - 13), were from hydrothermal fields, while the other species were from a brackish lake (14), a terrestrial mud volcano (15), seawater (16, 17), and marine sediments (18 - 21). No members of *Sulfurovum* and *Sulfurimonas* have been isolated from cold seeps. Both *Sulfurovum* spp. and *Sulfurimonas* spp. possess versatile energy metabolisms. They can use hydrogen sulfide, hydrogen, sulfur, and thiosulfate as electron donors and use nitrate, sulfur, thiosulfate, and oxygen as electron acceptors (12). Since it is challenging to obtain *in situ* undisturbed samples in deep-sea and extract high-quality RNA or protein, very few studies on the *in situ* metabolism of Campylobacterota in hydrothermal vents have been reported (22), and no such study on deep-sea cold seep Campylobacterota has been documented.

The Formosa cold seep is located in the South China Sea (SCS), where several active seeps have been found. In our previous cruises, a large number of macrobenthos were observed around the seepage, including crabs (*Shinkaia crosnieri*), mussels (*Bathymodiolus platifrons*), and shrimps (*Alvinocaris longirostris*), and a large area of black RS was also found (23). Our previous study demonstrated that Campylobacterota, especially *Sulfurovum*, was dominant in the macrobenthic area and the RS (23). The abundant distribution of Campylobacterota suggests that these bacteria play a critical role in the geochemical cycle of the cold seep. Therefore, the Formosa cold seep is an ideal place to investigate and understand the ecological function of Campylobacterota in cold seeps.

The aim of this study was to investigate the geochemical role and *in situ* function of Campylobacterota in deep-sea cold seep. For this purpose, we successfully isolated new species of *Sulfurovum* and *Sulfurimonas* from the Formosa cold seep and characterized their phenotypic and biochemical features. We then examined the ecological role of Campylobacterota on a comprehensive scale using multiple approaches, including comparative genomics, metatranscriptomics, geochemical analysis, and *in situ* experiments.

## MATERIALS AND METHODS

### Sampling and *in situ* incubation

Samples used in this study were collected from Formosa cold seep in the SCS during the scientific cruises of “Kexue” in 2020 and 2021. Sediment samples were obtained with a push core as previously reported (23). Porewater was collected from the sediment sample with a soil solution sampler (Rhizon, Holland). *S. crosnieri* was collected with a remotely operated vehicle. A gas-tight sampler based on a lander platform was used to collect seawater around the seepage. Raman probes were deployed near the sample inlet to detect the geochemical parameters in the gas-tight samples. Seepage-surrounding water was collected with the gas-tight sampler and placed *in situ* for 14 days and then used for analysis. As a control, the seepage-surrounding water was collected and analyzed without *in situ* incubation.

### Geochemical analysis

Hydrogen sulfide in the porewater was measured by the methylene blue method (24). The concentration of nitrate (NO_3_^−^) was analyzed colorimetrically with a Quaatro continuous flow analyzer (SEAL Analytical Ltd, Southampton, UK). Sulfate concentration was determined using a Dionex Ion Chromatograph (Thermo Fisher Scientific, Waltham, MA, USA). The ammonium concentration in the porewater was measured onboard via indophenol blue spectrophotometry (25).

### Bacterial enrichment and isolation

To enrich the Campylobacterota in the upper RS and *S. crosnieri* setae, each sample was transferred to 10-mL of anaerobic MJ medium (DSMZ 1011) in a sterile 15-mL centrifuge tube. After vibrating for 3–5 minutes, 0.5 mL of suspension was transferred to 100 mL of MJ medium in a glass bottle tightly sealed with butyl rubber under the condition of 80% H_2_/20% CO_2_ (200 kPa). The sample was maintained at 10℃ for 1 month. Bacteria was isolated by serial dilution. Two isolates were named CS14^T^ and CS47^T^. The purity of the isolates was verified by 16S rRNA gene sequencing, microscopic observation, and heterotrophic cultivation. The type strain *Sulfurovum denitrificans* DSM 19611^T^ was obtained from Deutsche Sammlung von Mikroorganismen und Zellkulturen GmbH (DSMZ, Braunschweig, Germany). The type strains *Sulfurovum lithotrophicum* JCM 12117^T^, *Sulfurovum riftiae* JCM 30810^T^, *Sulfurimonas autotrophica* JCM 11897^T^, and *Sulfurimonas gotlandica* JCM 16533^T^ were obtained from Japan Collection of Microorganisms (JCM, Ibaraki, Japan). These strains were used as reference strains for fatty acid methyl ester (FAME) analysis and phenotypic characterization.

### Phenotypic, phylogenetic, and chemotaxonomic analysis

The cellular morphology of the strains was observed with a transmission electron microscope (HT7700; Hitachi, Tokyo, Japan). Gram staining was performed with Gram staining kit (Haibo, Qingdao, China). To examine the temperature range of growth, the bacterium was grown in MJ medium under 80% H_2_/20% CO_2_ at 0°C, 5°C, 10°C, 15°C, 20°C, 25°C, 28°C, 30°C, 35°C, 37°C, 40°C, and 45°Cfor 2 weeks, and the OD_600_ (optical density at 600 nm) value of the bacterial solution was measured. To examine the NaCl range of growth, the bacterium was grown in MJ medium containing different concentrations of NaCl (wt/vol, 0%–10%, with an interval of 1%) at the optimal temperature and under the condition of 80% H_2_/20% CO_2_. To examine the pH range of growth, the bacterium was grown in MJ medium under 80% H_2_/20% CO_2_ at the optimal temperature with different buffers (12). Doubling time was assessed according to a previous report (26).

Utilization of electron donor was tested in MJ medium under N_2_/CO_2_. The following potential electron donors were used: Na_2_S (2.5 µM, 5 µM, 10 µM, 20 µM, 40 µM, 60 µM, 80 µM, 100 µM, 500 µM, 1 mM, and 2 mM), Na_2_S_2_O_3_ (5, 10, 20, 40, 60, 80, and 100 mM), Na_2_SO_3_ (5 mM, stocked in 2 mM Na_2_EDTA), Na_2_S_4_O_6_ (5 mM), and elemental S (S^0^, 5 g/L). For the test of hydrogen as the electron donor, 80% H_2_/20% CO_2_ was used. Utilization of electron acceptor was tested in MJ medium under 80% H_2_/20% CO_2_. The following potential electron acceptors were tested: NaNO_3_ (23.5 mM), S^0^ (5 g/L), Na_2_S_2_O_3_ (5 mM), and O_2_ (1%, 2%, 3%, 5%, 7%, and 10% vol/vol). Heterotrophic growth was tested in MJ medium without NaHCO_3_ under 80% H_2_/20% N_2_, with the following organic substances used: (1) monosaccharide solution (0.1% wt/vol): glucose, fructose, and galactose; (2) disaccharide solution (0.1% wt/vol): maltose, lactose, sucrose, and trehalose; (3) yeast powder (0.1% wt/vol); (4) peptone (0.1% wt/vol); (5) organic acid solution (5 mM): acetic acid, malic acid, fumaric acid, and succinic acid; and (6) 20 amino acid solutions (1 mM).

Phylogenetic analysis was conducted according to our previous report (27). The phylogenomic tree was reconstructed based on an up-to-date 92 bacterial core gene sets by UBCG version 3.0 (21). For the analysis of FAME, bacteria were grown at the optimum temperature and harvested after 7–10 days of growth. Data for FAME were analyzed as previously reported (28).

### Amplicon analysis, genomics, and metatranscriptomics

Amplicon sequencing and analysis were performed as previously reported (23). Bacterial genome sequencing and analysis were performed according to our previous report (27). The functions of the genes were annotated with NCBI-NR and KEGG databases. For genome comparison, 18 *Sulfurovum* and *Sulfurimonas* genomes were obtained from NCBI (Table S1). Three *S*. *crosnieri* setae samples and one top surface sample of RS were used for metatranscriptomic sequencing. RNA was extracted with E.Z.N.A. Soil RNA Kit (Omega Bio-Tek, Norcross, GA, USA). The cDNA library of each sample was constructed and sequenced with the HiSeq 3000 platform (Illumina, San Diego, CA, USA). Adapters and low-quality reads (base quality ≤ 20) were trimmed with Cutadapt. The clean reads were assembled, and the genes were predicted with megahit (29) and MetaGeneMark (30). All predicted genes were filtered by 100-bp length cutoff, and the redundancies were removed by cd-hit. Transcript expression levels were quantified and expressed fragments per kilobase of transcript per million fragments mapped (FPKM) (31). Metatranscriptome catalogs were searched against the NR database to obtain the taxonomy and function of genes, and genes belonged to *Sulfurovum* and *Sulfurimonas* were used to conduct expression analyses.

## RESULTS

### *Sulfurovum* and *Sulfurimonas* are abundant in the upper reduced sediments

**Fig 1:**
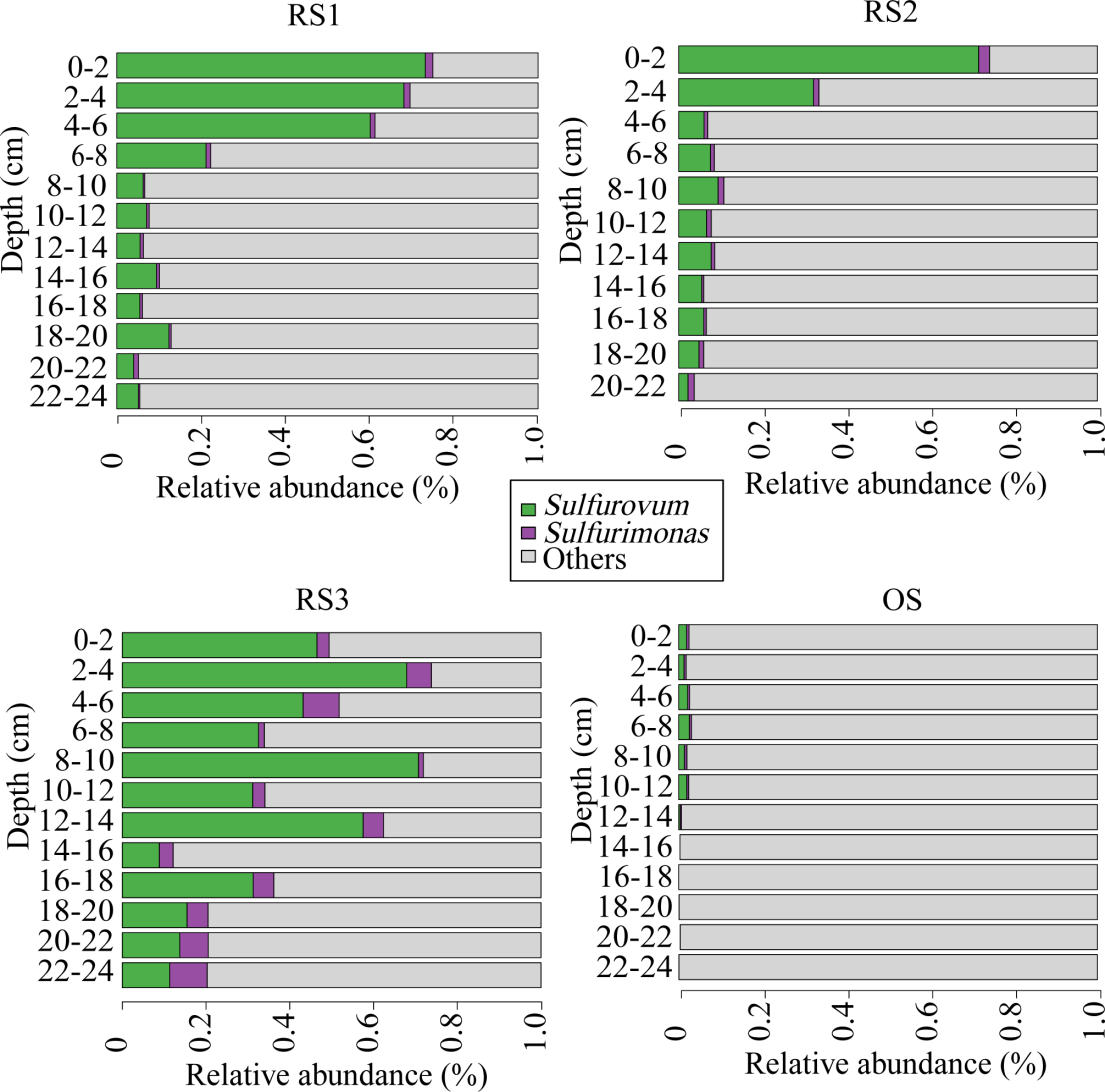
The diversity and distribution of microbial sequence tags in the libraries of the sediment samples of RS1 to 3 and OS. The numbers indicate the depth (centimeter) of the samples. The sequence tags were classified at the genus level. *Sulfurovum* and *Sulfurimonas* are indicated, and all other genera are integrated as “Others.”

Four sediment samples from the Formosa cold seep were used for microbial community analysis. Of these samples, three were collected from the RS zone and one (the control) was collected from the oxidative sediment (OS) zone. Amplicon analysis indicated that, in reduced sediment 1 (RS1), *Sulfurovum* was abundant at depths of 0–8 cm and 18–20 cm, accounting for 12.4% to 73.2% of the microbial libraries, and was less abundant at other depths, accounting for 4.0%–9.4% of the libraries (Fig. 1). In reduced sediment 2 (RS2), *Sulfurovum* was rich at 0–4 cm (32.2%–71.6% in abundance) and relatively low at other depths (2.2%–9.4% in abundance) (Fig. 1). In reduced sediment 3 (RS3), *Sulfurovum* accounted for 8.8%–70.7% at all depths and had an abundance of more than 50% at 2–4 cm, 8–10 cm, and 12–14 cm (Fig. 1). *Sulfurimonas* represented a very small proportion at all depths of RS1 (0.4%–1.8%) and RS2 (0.5%–2.6%) (Fig. 1). In RS3, *Sulfurimonas* was relatively abundant at 2–6 cm and 18–24 cm (>5.0%), and its abundance was low at other depths (Fig. 1). In OS, *Sulfurovum* and *Sulfurimonas* were very low in all depths, accounting for 0%–2.6% and 0%–0.6%, respectively, in abundance (Fig. 1).

### Microbe-mediated nitrate consumption in the reduced sediment–water interface leads to a sharp decrease in the nitrate level

**Fig 2:**
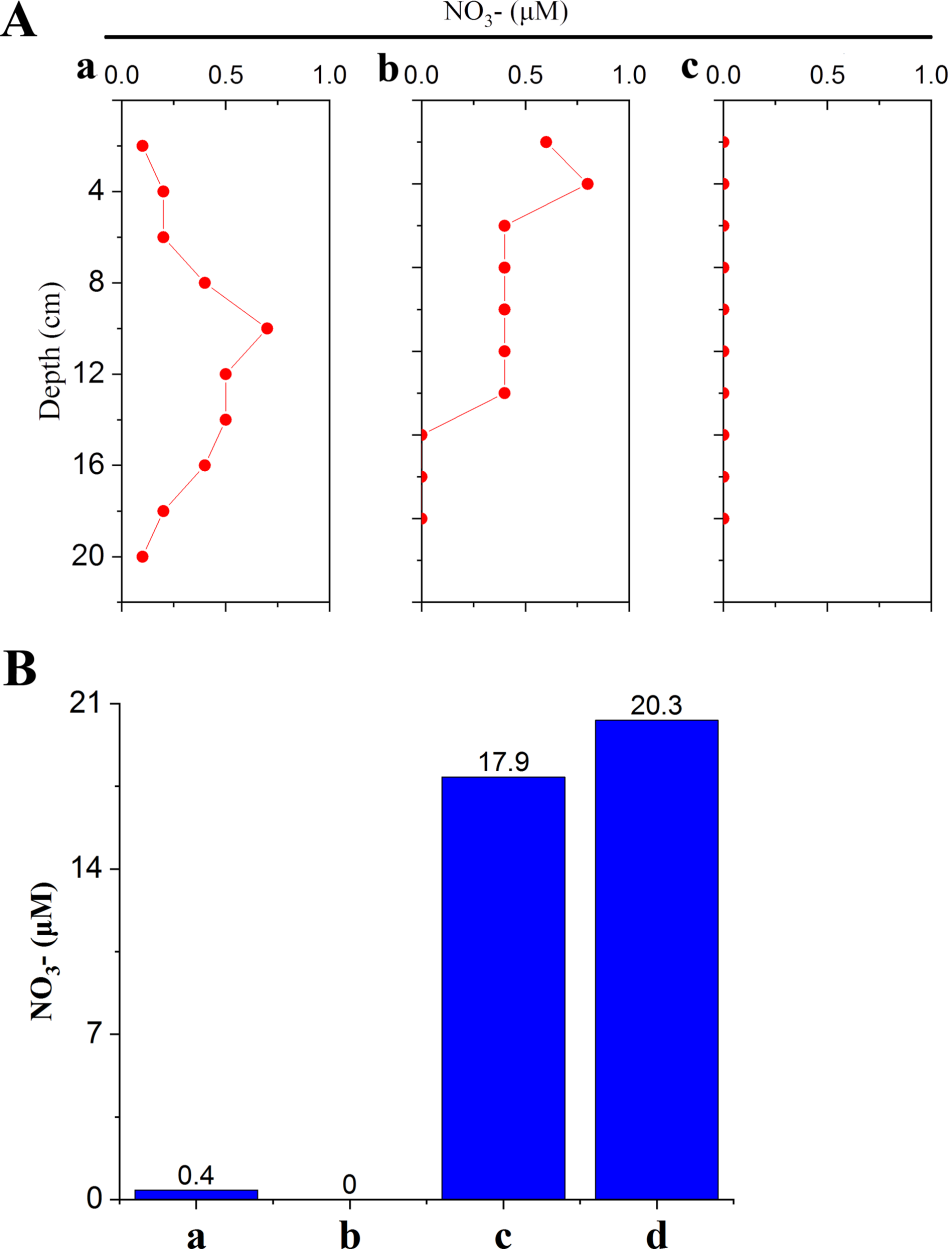
Nitrate level in reduced sediments and the sediment–water interface. (A) Nitrate concentration in sediment samples 1 to 3 (a to c) at different depths was determined. (B) Seepage-surrounding water in a gas-tight sampler was placed *in situ* for 14 days (a and b) and then determined for nitrate concentration. As the control, the nitrate concentration in the seepage-surrounding water without *in situ* incubation was determined (c and d).

In the top surface of RS1–3, the hydrogen sulfide concentration ranged from 0.8 to 3.9 mM, the ammonia concentration ranged from 61 to 163 µM, and the sulfate concentration ranged from 19.1 to 27.7 mM. In the top surface of OS, the hydrogen sulfide, ammonia, and sulfate concentrations were 0.02 mM, 26 µM, and 28.3 mM, respectively. Thiosulfate was also detected in the top surface of RS1–3 (26.7–705.4 µM) but not in that of OS. The nitrate concentration was 24.3 µM in the seawater close to the RS and plunged to very low levels in the sediments, such as a few hundred nanomoles in the subsurface and non-detectable in the deeper depths (Fig. 2A). An *in situ* experiment with seawater collected from the seepage vicinity showed that when the water was placed *in situ* in a gas-tight container for 14 days, its nitrate level was reduced by nearly 100% (Fig. 2B). Because nitrate-reducing *Sulfurovum* is rich in these seawaters (23), these results suggest that nitrate consumption by microbes in the seawater close to the seepage led to a sharp reduction in the nitrate level.

### Isolation of new species of *Sulfurovum* and *Sulfurimonas* from the Formosa cold seep

#### Morphological, phenotypic, and chemotaxonomic characterization

Since, as shown above, *Sulfurovum* and *Sulfurimonas* are abundant in the upper RS, but neither *Sulfurovum* nor *Sulfurimonas* has been isolated from cold seeps, we endeavored to isolate members of these genera. We succeeded in the isolation of two strains, CS14^T^ and CS47^T^, from the Formosa cold seep. Strain CS14^T^ was Gram-stain-negative, ellipsoidal or short rod-shaped, anaerobic, 0.8–1.0 μm in width and 1.5–3.0 μm in length, and lacked flagella (Fig. S1). CS14^T^ could grow in a range of temperatures (5℃–20℃), salinities (2%–4% [wt/vol]), and pH levels (5.5–8.5), with the best growth occurring at 10℃, 3% NaCl (wt/vol), and pH 6.5 (Table 1). The doubling time of CS14^T^ was approximately 25 hours. When nitrate was used as the electron acceptor, strain CS14^T^ grew in a chemoautotrophic manner only with H_2_ as an electron donor and did not grow with sulfide, thiosulfate, sulfite, ditetrasulfate, or S^0^ as an electron donor (Table 1). With hydrogen as an electron donor, CS14^T^ could grow with nitrate as an electron acceptor but did not grow with thiosulfate, S^0^, or oxygen as an electron acceptor (Table 1). Heterotrophic growth showed that strain CS14^T^ did not grow in the tested organic compounds (Table 1), indicating that CS14^T^ was chemoautotrophic. Strain CS47^T^ was Gram-stain-negative, slightly curved, facultatively anaerobic, 0.3–0.5 μm in width, and 1.5–2.5 μm in length with a single polar flagellum (Fig. S1). Strain CS47^T^ grew in a range of temperatures (0°C–37°C), salinities (1%–6% [wt/vol]), and pH levels (6.0–7.5), with optimal growth at 25°C–28°C, 2%–3% NaCl (wt/vol), and pH 6.5 (Table 2). The doubling time of CS47^T^ was approximately 7 hours. With nitrate as the electron acceptor, strain CS47^T^ could grow with H_2_ or elemental sulfur as an electron donor (Table 2). When hydrogen was used as the electron donor, CS47^T^ could grow with nitrate or oxygen (no more than 7%) as an electron acceptor (Table 2). Heterotrophic growth showed that like strain CS14^T^, CS47^T^ was chemoautotrophic and did not grow in the tested organic compounds (Table 2). FAME analysis showed that, similar to the reference strains, the major fatty acids (≥ 5%) of strain CS14^T^ contained C_16:1_ ω7c (48.8%), C_16:0_ (12.7%), C_14:0_ (12.3%), and C_12:0_ (9.2%); summed features 10 (8.9%); and summed features 5 (5.5%) (Table S2). The major fatty acids (≥ 5%) of strain CS47^T^ contained C_16:1_ ω7c (49.4%), C_16:0_ (24.5%), summed feature 10 (8.9%), C_14:0_ (8.1%), and summed feature 5 (7.8%), which were similar to the major fatty acids of the reference strains (Table S2).

**TABLE 1:** Comparison of the characteristics of *Sulfurovum fonticola* CS14^T^ sp. nov. with the related species of the genus *Sulfurovum^a^*

Characteristics	1*	2	3	4	5	6
Shape, motility	Ellipsoid or short rods, −	Rods, −	Rods, −	Coccoid to oval, −	Rods, +	Coccoid to oval, −
Temperature range (℃) (optimal)	5–20 (10)	15–37 (33)	10–35 (30)	10–40 (28–30)	25–40 (35)	4–50 (37)
pH range (optimal)	5.5–8.5 (6.5)	5.5–8.6 (6.0)	6.5–7.5 (7.0)	4.5–9.0 (6.5–7.0)	5.0–8.0 (6.0)	5.0–8.6 (6.0)
NaCl range (% wt/vol) (optimal)	2.0–4.0 (3)	2.0–4.0 (2.5)	2.0–5.0 (3.0)	1.0–6.0 (4.0)	1.5–4.0 (3.0)	1.0–5.0 (3.0)
Doubling time under optimal conditions (hours)	25	3.2	ND	1.5	3.0	3.2
Electron donor	H_2_	H_2_	H_2_, S^0^, S_2_O_3_^2−^*	S^0^, S_2_O_3_^2−^*	H_2_, S^0^, S_2_O_3_^2−^*	H_2_, HS^−^, S^0^, S_2_O_3_^2−^
Heterotrophic growth	−	−	−*	−*	−*	−
Organic electron donor	−	−	−	−	−	−
Electron acceptor	NO_3_^−^	NO_3_^−^, S^0^, S_2_O_3_^2−^	NO_3_^−^, O_2_*	NO_3_^−^, O_2_*	NO_3_^−^, O_2_*	NO_3_^−^, S^0^, O_2_
G + C content (mol%)	37.6	42.6	40.0	48.0	47.4	42.4

^*a*^ 1, *Sulfurovum fonticola* CS14^T^; 2, *Sulfurovum aggregans* Monchim33^T^ (7); 3, *Sulfurovum denitrificans* DSM 19611^T^ (5); 4, *Sulfurovum lithotrophicum* JCM 12117^T^ (8); 5, *Sulfurovum riftiae* JCM 30810^T^ (6); 6, *Sulfurovum indicum* ST-419^T^ (4). *data from this study. ND, not determined; +, positive; −, negative.

**TABLE 2:** Comparison of the characteristics of *Sulfurimonas fonticola* CS47^T^ sp. nov. with related species of the genus *Sulfurimonas^a^*

Characteristics	1	2	3	4	5	6	7	8	9	10	11	12	13	14	15	16
Shape motility	Rods to slightly curved, +	Curved rods to spirilla-like, +	Rods, +	Rods to slightly curved, +	Rods to slightly curved, +	Rods, +	Rods, +	Rods, +	Rods, +	Rods, +	Rods, +	Rods to slightly curved, +	Rods, +	Rods to slightly curved, –	Rods to spirilla-like, –	Rods, ND
Temperature range (optimal) (℃)	0–37(25–8)	4–20(15)	10–40 (23–26)	0–25 (20)	0–20(15)	3–22 (13–5)	4–40(33)	4–35 (30)	4–45(33)	10–45(33)	10–45(35)	4–45(33)	5–40(30)	10–45(30)	10–30(22)	15–35(30)
pH range (optimal)	6.5–7.5 (6.5)	6.5–8.4 (6.7–8.0)	4.5–9.0 (6.0–6.5)	6.5–9.0 (7.5–8.0)	7.0–8.0 (7.0–7.5)	6.0–7.4 (6.6–6.8)	4.5–7.5(5.5)	5.4–8.6(6.1)	5.0–9.0 (6.0–6.5)	5.0–8.0(7.0)	4.5–9.0(7.0)	5.0–8.5(6.5)	5.5–9.5(8.0)	5.5–8.0(7.0)	ND(7.0)	6.5–8.5(7.0–7.5)
NaCl range (optimal) (%）	1–6(2–3)	ND(1–2)	1.6–6(4)	1–4(2.5)	0.4–4(1.4)	2–5(2–3)	340–680 mM (510 mM)	1.2–5(3)	2–4(3)	1.5–6(3)	1–8(3)	0.5–9(2)	0.5–8(2–3)	0–9(5)	ND	1–6(3)
Doubling time under optimal conditions (hours)	7	13	1.4	9	6	ND	9	13–16	6	5	3.6	8	2.2	12	12	6.1
Electron donors	H_2_, S^0^	H_2_, S_2_O_3_^2−^*	S^0^, S_2_O_3_^2−^*	H_2_, HS^−^, S^0^, S_2_O_3_^2−^	H_2_, HS^−^, S^0^, S_2_O_3_^2−^	HS^−^, S^0^, S_2_O_3_^2−^	H_2_, HS^−^, S^0^, S_2_O_3_^2−^	H_2_, S^0^, S_2_O_3_^2−^	H_2_, HS^−^, S^0^, S_2_O_3_^2−^	H_2_, HS^−^, S^0^, S_2_O_3_^2−^, S_4_O_6_^2−^	HS^−^, S^0^, S_2_O_3_^2−^	H_2_, HS^−^, S^0^, S_2_O_3_^2−^	HS^−^, S^0^, S_2_O_3_^2−^	H_2_, HS^−^, S^0^, S_2_O_3_^2−^, S_4_O_6_^2−^	H_2_, HS^−^, S_2_O_3_^2−^	H_2_, HS^−^, S_2_O_3_^2−^
Heterotrophic growth	–	–*	–*	–	Short-chain fatty acids acetate, succinate, propionate mix	–	–	–	–	–	–	–	–	–	–	–
Organic electron donors	–	Formate, acetate, yeast extract, pyruvate, amino acid	–	–	–	–	–	–	–	–	–	–	–	–	Formate, fumarate, yeast extract, alcohol mix	–
Electron donors	NO_3_^−^, O_2_	NO_3_^−^, O_2_*	NO_3_^−^, O_2_*	MnO_2_, NO_3_^−^, O_2_	MnO_2_, NO_3_^−^, O_2_	O_2_	O_2_	NO_3_^−^, O_2_	NO_3_^−^, O_2_, S^0^	NO_3_^−^, O_2_, S^0^	O_2_	NO_3_^−^, O_2_, S^0^	NO_3_^−^, NO_2_^-^, O_2_	NO_3_^−^, O_2_, S^0^	NO_3_^−^, NO_2_^−^, O_2_	NO_3_^−^
G + C content (mol%)	32.1	33.6	35.2	33.5	33.8	34.7	36.9	37.6	37.3	37.3	36.0	33.2	38.8	34.5	36.0	34.9

^*a*^ 1, *Sulfurimonas fonticola* CS47^T^; 2, *Sulfurimonas gotlandica* JCM 16533^T^ (16); 3, *Sulfurimonas autotrophica* JCM 11897^T^ (10); 4, Candidatus *Sulfurimonas marisnigri* SoZ1 (17); 5, Candidatus *Sulfurimonas baltica* GD2 (17); 6, *Sulfurimonas aquatica* H1576^T^ (14); 7, *Sulfurimonas indica* NW8N^T^ (9); 8, *Sulfurimonas paralvinellae* GO25^T^ (11); 9, *Sulfurimonas hydrogeniphila* NW10^T^ (12); 10, *Sulfurimonas sediminis* S2-6^T^ (13); 11, *Sulfurimonas marina* B2^T^ (21); 12, *Sulfurimonas lithotrophica* GYSG_1^T^ (18); 13, *Sulfurimonas crateris* SN118^T^ (15); 14, *Sulfurimonas xiamenensis* 1–1N^T^ (18); 15, *Sulfurimonas denitrificans* DSM 1251^T^ (11, 20); 16, *Sulfurimonas hongkongensis* AST-10^T^ (19). * data from this study; ND, not determined; +, positive; −, negative.

#### Phylogenetic and phylogenomic analysis

**Fig 3:**
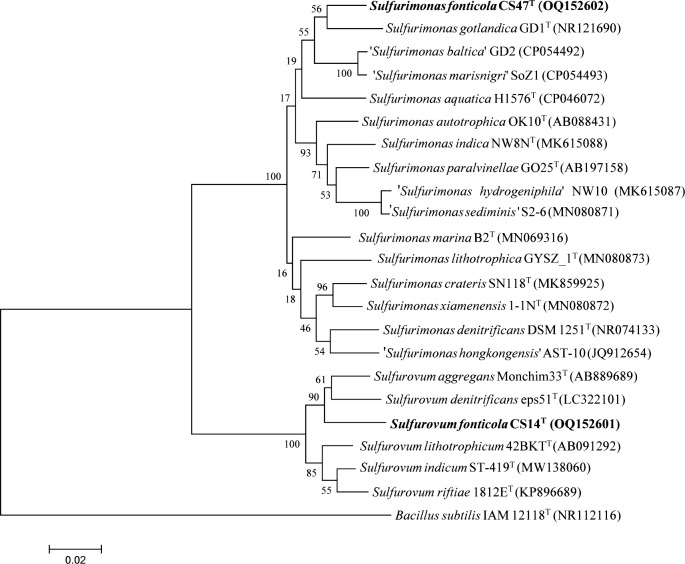
Phylogenetic analysis of strains CS14^T^ and CS47^T^. The neighbor-joining phylogenetic tree based on 16S rRNA gene sequence shows the positions of *Sulfurovum fonticola* CS14^T^, *Sulfurimonas fonticola* CS47^T^, and representatives of other related taxa. *Bacillus subtilis* IAM12118^T^ (GenBank accession no. NR112116) was used as an outgroup. Scale bar, 0.02 substitution per nucleotide position.

The analysis of the 16S rRNA gene sequence indicated that strain CS14^T^ belongs to the genus *Sulfurovum* and shares the highest sequence identity with *Sulfurovum aggregans* Monchim33^T^ (96.3%). The identities between strain CS14^T^ and other close members of *Sulfurovum* are below 96.0%. These levels of identities are below the suggested threshold for bacterial species delineation (32). Based on the 16S rRNA gene sequence analysis, strain CS47^T^ belongs to the genus *Sulfurimonas* and shares the highest sequence identities with *Sulfurimonas gotlandica* GD1^T^ (96.2%) and *Sulfurimonas hongkongensis* AST-10^T^ (96.1%). The identities between strain CS47^T^ and other close members of *Sulfurimonas* are below 95.0%. Phylogenetic analysis showed that strain CS14^T^ formed a group with *Sulfurovum* spp. and represented a basal member of this group (Fig. 3). Strain CS47^T^ formed a group with *Sulfurimonas* spp., further supporting the proposal that strain CS47^T^ belongs to the genus *Sulfurimonas* (Fig. 3). Similar results were observed in the phylogenomic analysis (Fig. S2). Genomic analysis indicated that strain CS14^T^ contains a circular chromosome of 2,753,309 bp with 2,754 predicted genes, and strain CS47^T^ contains a circular chromosome of 2,596,819 bp with 2,531 predicted genes. The G + C contents of the CS14^T^ and CS47^T^ genomes are 37.6% and 32.1%, respectively (Tables 1 and 2). The average nucleotide identity (ANI) values and the digital DNA–DNA hybridization (DDH) values between strain CS14^T^ and its close members in *Sulfurovum* are listed in Table S3. The ANI values and the digital DDH values between strain CS47^T^ and its close members in *Sulfurimonas* are listed in Table S3. All of these ANI and DDH values are much lower than the threshold values for prokaryotic species delineation (95%–96% for ANI and 70% for DDH) (33, 34). Together, these results indicate that strains CS14^T^ and CS47^T^ represent novel species of *Sulfurovum* and *Sulfurimonas*, respectively.

### Comparative genomic analyses reveal a genetic basis for the versatile metabolisms of *Sulfurovum* and *Sulfurimonas*

#### Hydrogen oxidation

**Fig 4:**
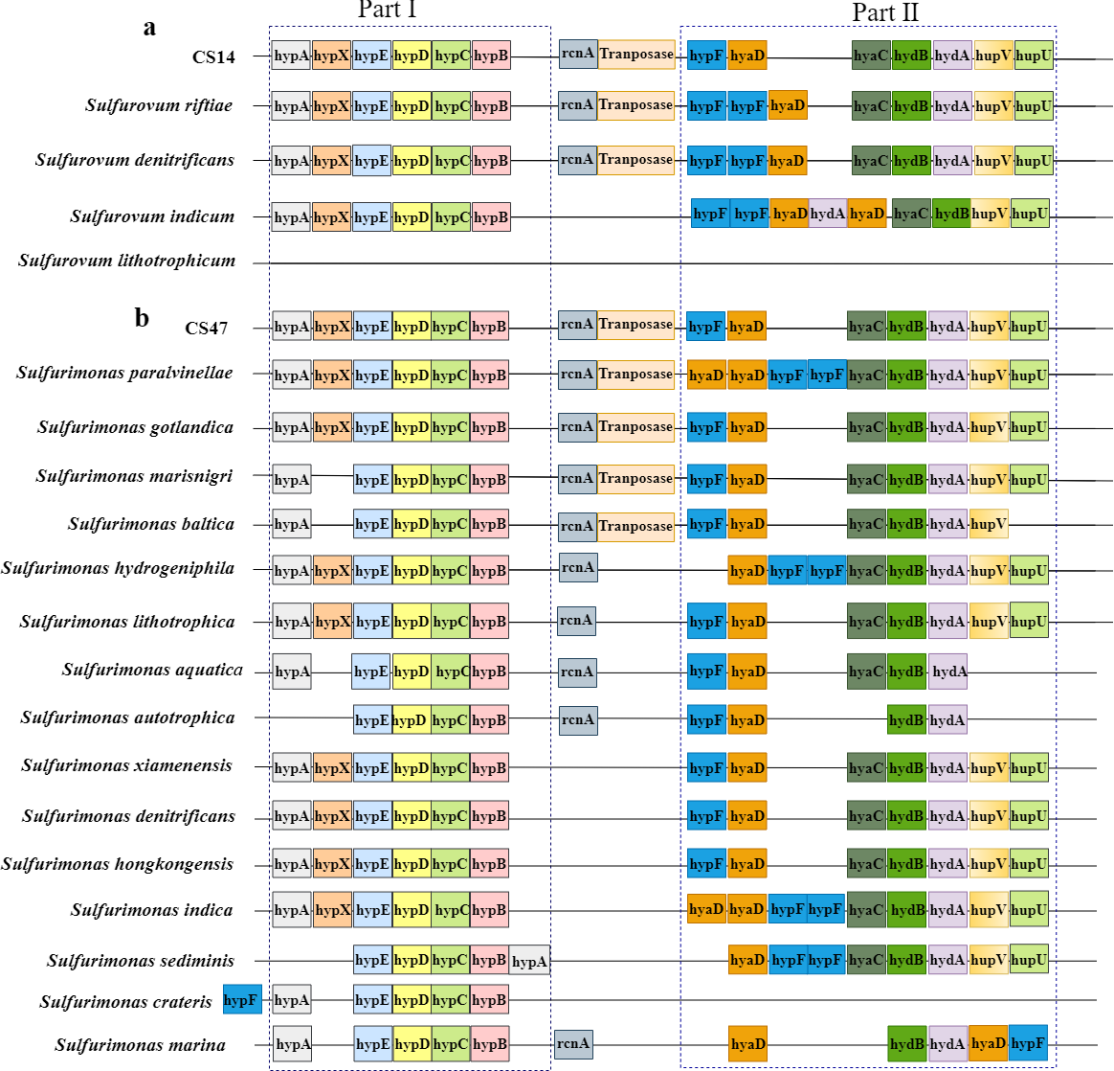
Comparison of the hydrogen-oxidizing clusters in *Sulfurovum* (**a**) and *Sulfurimonas* (**b**) based on KEGG annotation.

To investigate the metabolic mechanism of *Sulfurovum* and *Sulfurimonas* at the genetic level, comparative genomic analyses were performed. A cluster of hydrogen oxidizing-associated genes was found in the genomes of *Sulfurovum* and *Sulfurimonas*. This cluster consists of two parts: Part I containing *hypAEDCB* (encoding hydrogenase nickel incorporation protein and hydrogenase expression/formation protein) with or without *hypX* (encoding hydrogenase maturation factor) and Part II containing *hypF* (encoding hydrogenase maturation protein), *hyaD* (encoding hydrogenase maturation protease), *hyaC* (encoding Ni/Fe-hydrogenase 1 B-type cytochrome subunit), *hydBA* (encoding [NiFe] hydrogenase large and small subunits), and *hupUV* (encoding uptake hydrogenase large and small subunits) (Fig. 4). *Sulfurovum* spp. with the ability of hydrogen oxidation possess the complete cluster, while *Sulfurovum lithotrophicum*, which is unable to use hydrogen as an electron donor, is devoid of this cluster (Fig. 4). All *Sulfurimonas* capable of hydrogen oxidation possess the complete cluster, except for *Sulfurimonas baltica*, which lacks *hupU* (Fig. 4). Four *Sulfurimonas* spp. unable to use hydrogen as energy possess incomplete hydrogen-oxidizing cluster: *Sulfurimonas crateris* lacks almost the entire part II; *S. autotrophica* lacks *hypA*, *hyaC*, and *hupUV*; and *Sulfurimonas aquatic* and *Sulfurimonas marina* lack *hupUV* (Fig. 4).

#### Sulfur oxidation

All *Sulfurovum* spp. possess two sulfur-oxidizing systems, that is, *soxABXYZ* and *soxCDYZ*, except for strain CS14^T^ isolated in this study, which only possesses *soxCDYZ* (Table S4). For *Sulfurimonas* spp., most possess two sulfur-oxidizing systems, except for the strain CS47^T^, *Sulfurimonas xiamenensis* and *Sulfurimonas lithotrophica*, which only have *soxCDYZ* (Table S4). All *Sulfurovum* spp. and *Sulfurimonas* spp. have sulfide:quinone oxidoreductase (*sqr*) with variation in number (Table S4).

#### Nitrogen metabolism

All *Sulfurovum* spp. have the denitrification pathway but lack the assimilation nitrate reduction, dissimilation nitrate reduction, and nitrogen fixation pathways (Table S4). Nitrogen metabolism in *Sulfurimonas* spp. is complex. *S. autotrophica*, *S. crateris*, *Sulfurimonas denitrificans*, *S. gotlandica*, *S. hongkongensis*, *S. paralvinellae*, *S. xiamenensis*, *S. sediminis*, *S. hydrogeniphila*, and *S. indica* only possess the denitrification pathway (Table S4). *S. aquatica* only possesses assimilation nitrate reduction pathway (Table S4). Candidatus *Sulfurimonas marisnigri* possesses assimilation nitrate reduction and nitrogen fixation pathways (Table S4). CS47^T^ and *S. lithotrophica* possess denitrification, assimilation nitrate reduction, and nitrogen fixation pathways (Table S4). *S. baltica* possesses dissimilation nitrate reduction, assimilation nitrate reduction, and nitrogen fixation pathways. *S. marina* only possesses nitrogen fixation pathways.

### Metatranscriptomic analysis unveils the *in situ* metabolic activities of *Sulfurovum* and *Sulfurimonas*

**Fig 5:**
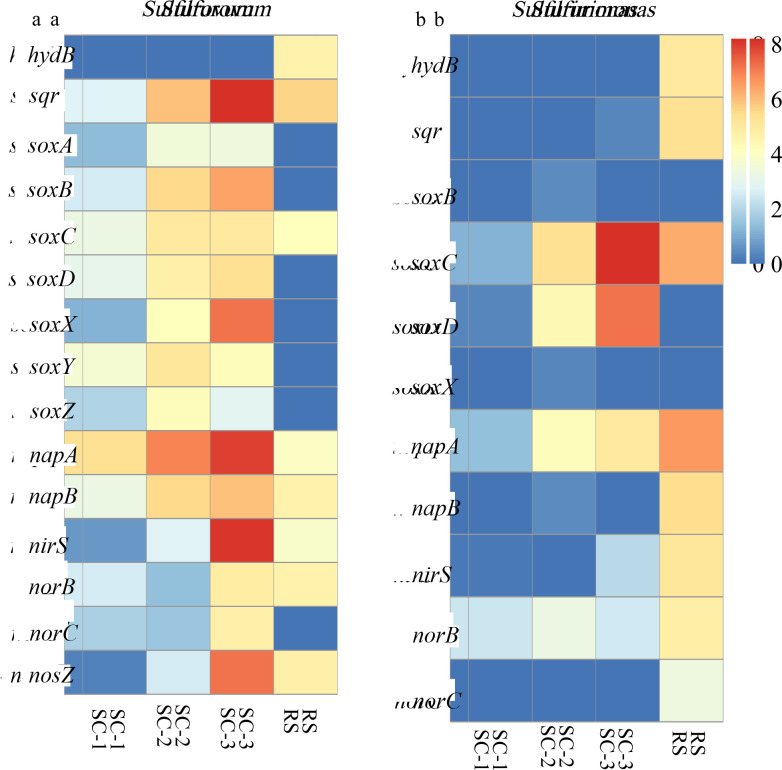
Heat maps of the genes associated with energy metabolism in *Sulfurovum* (**a**) and *Sulfurimonas* (**b**). Transition from blue to red represents increased expression. SC-1 to 3 are shrimp setae; RS is reduced sediments. Fragments per kilobase of transcript per million fragments mapped (FPKM) is presented in log10 scale, and the FPKM values are listed in Table S5 and S6.

To understand the *in situ* metabolism of *Sulfurovum* and *Sulfurimonas*, metatranscriptomic analysis was conducted. Given the difficulty of using seepage seawater for metatranscriptomics and the fact that *Sulfurovum* is abundant in the setae of the shrimp (*S. crosnieri*) inhabiting the seepage surroundings (23), we included three samples of shrimp setae (SC-1 to 3) in the metatranscriptomic analysis. The results showed that the hydrogen oxidization–associated gene *hydB* of *Sulfurimonas* and *Sulfurovum* was expressed at high levels in the surface of RS (153.5 and 111.6 in FPKM, respectively), but hydrogen oxidization–associated gene expression was not found in SC-1 to 3 (Fig. 5; Table S5 and S6). The sulfide-oxidizing gene *sqr* of *Sulfurovum* was expressed at high levels in SC-1 to 3, especially in SC-3 (4061.6 in FPKM), and on the surface of RS (302.9 in FPKM) (Fig. 5; Table S5). The expression of *Sulfurimonas sqr* was also high in the surface of RS (214.6 in FPKM) but very low or undetectable in *S. crosnieri* setae (Fig. 5; Table S6). The *soxABCDXYZ* genes of *Sulfurovum* were expressed at a high level in *S. crosnieri* setae (especially in SC-2 and SC-3) (Fig. 5; Table S5), indicating that two sulfur-oxidizing systems (*soxABXYZ* and *soxCDYZ*) were operating in *S. crosnieri* setae. On the surface of RS, only *soxC* of *Sulfurovum* was expressed at a high level (63.7 in FPKM) (Fig. 5; Table S5). For *Sulfurimonas*, *soxCD* genes were expressed at a high level in *S. crosnieri* setae, especially in SC-2 and SC-3 (FPKM > 1,000), *soxC* was expressed at a high level in the surface of RS (529.9 in FPKM), and *soxABX* was not expressed or was expressed at an extremely low level (FPKM < 1.0) in *S. crosnieri* setae and on the surface of RS (Fig. 5; Table S6). These results indicated that only one sulfur-oxidizing system (*soxCDYZ*) of *Sulfurimonas* was working in the RS and *S. crosnieri* setae. All of the denitrification-associated genes of *Sulfurovum* were expressed at high levels in shrimp setae and on the surface of RS (Fig. 5; Table S5), except for *norC*, which was not detected on the surface of RS. For *Sulfurimonas*, all of the denitrification-associated genes were expressed at high levels on the surface of RS, except for *nosZ*, whose expression was not detected (Fig. 5; Table S6). *NapA* and *norB* were expressed at high levels in *S. crosnieri* setae, while other denitrification genes were not expressed or were expressed at low levels (Fig. 5; Table S6). For carbon fixation, previous reports indicated that Campylobacterota could fix carbon via the reductive tricarboxylic acid pathway (35). Indeed, all of the genes of the reductive tricarboxylic acid pathway were found in the genomes of CS14^T^ and CS47^T^. The key enzyme of this cycle, ATP-dependent citrate lyase, was also found in all of the analyzed genomes of *Sulfurovum* and *Sulfurimonas*. Metatranscriptome analyses indicated that ATP-dependent citrate lyase of *Sulfurovum* was expressed at high levels in all samples (Table S5). In addition, two transcripts encoding nitrogenase belonging to anaerobic methane-oxidizing archaea (ANME) and methanogenic archaea were expressed at high levels in the RS (Table S7).

## DISCUSSION

Although *Sulfurimonas* and *Sulfurovum* are typical autotrophic bacteria dominant in both deep-sea hydrothermal vents and cold seeps, they have been isolated only from hydrothermal vents. In this study, two members of *Sulfurovum* and *Sulfurimonas* were isolated for the first time from a cold seep. Like most cultivable *Sulfurovum* spp. and *Sulfurimonas* spp. from other environments, the two cold seep isolates can use hydrogen as an electron donor and possess a hydrogen-oxidizing cluster in their genomes. Comparative genomic analysis indicated that this cluster is complete in all but one examined *Sulfurovum* spp. and *Sulfurimonas* spp. that can oxidize hydrogen and is incomplete in *Sulfurovum* spp. and *Sulfurimonas* spp. that cannot use hydrogen. These results indicated that this gene cluster is probably essential to hydrogen oxidation. A previous study has reported that hydrogen can serve as an energy source in hydrothermal vents (36). It is unknown whether hydrogen can also be an energy source in deep-sea cold seeps. In this study, both strain CS14^T^ and strain CS47^T^ isolated from a cold seep used hydrogen as the electron donor, and metatranscriptomic analysis demonstrated the high expression of hydrogen-oxidizing genes in the RS. These results suggest that hydrogen is likely to serve as an energy source in deep-sea cold seeps.

In addition to hydrogen, hydrogen sulfide is an important energy source in deep-sea hydrothermal vents as well as cold seeps. Previous studies have shown that most *Sulfurimonas* spp. and one *Sulfurovum* can use sulfide as the electron donor (4, 12). In this study, it was found that all *Sulfurimonas* and *Sulfurovum* sp. possess the sulfide-oxidizing gene (*sqr*). Although the isolates CS14^T^ and CS47^T^ could not use sulfide under laboratory conditions, the *sqr* gene of *Sulfurovum* and *Sulfurimonas* was expressed at high levels in RS, suggesting that *Sulfurovum* and *Sulfurimonas* used sulfide as an electron donor *in situ*. Further, comparative genome analysis indicated that most *Sulfurovum* and *Sulfurimonas* have two sulfur-oxidizing systems (*soxABXYZ* and *soxCDYZ*), and only a small number of these bacteria, including CS14^T^ and CS47^T^ isolated in this study, have one sulfur-oxidizing system (*soxCDYZ*). Differences in the number of the sulfur-oxidizing system can cause differences in metabolic features. When sulfide was the sole electron donor, *Sulfurimonas* with *soxABXYZ* and *soxCDYZ* could completely oxidize sulfide to sulfate, while *Sulfurimonas* with *soxCDYZ* alone could only partially oxidize sulfide to form thiosulfate and sulfate (37). In this study, high concentrations of sulfide and thiosulfate were detected in RS, in which only the *soxCDYZ* system was expressed at a high level, suggesting that partial oxidation of sulfide in the RS contributed to thiosulfate accumulation. In contrast, both *soxABXYZ* and *soxCDYZ* of *Sulfurovum* were expressed at high levels in the microbial community of shrimp setae, implying complete oxidation of sulfide to sulfate.

Previous studies have demonstrated that the denitrification pathway is common in *Sulfurovum* and *Sulfurimonas* (12), and a recent study has shown that two *Sulfurimonas* species are capable of nitrogen fixation (17). In the present study, we systematically examined the nitrogen metabolism pathways of *Sulfurovum* spp. and *Sulfurimonas* spp. via comparative genomic analysis. We found that all examined *Sulfurovum* spp. have the denitrification pathway, while *Sulfurimonas* spp. have versatile nitrogen metabolisms, including denitrification, dissimilatory denitrification reduction, assimilation denitrification reduction, and nitrogen fixation pathway, of which, the last three were identified for the first time in *Sulfurimonas* genomes. In the metatranscriptome, denitrification-associated genes of *Sulfurovum* and *Sulfurimonas* were expressed at high levels in RS and shrimp seta microbiota, indicating an active denitrification process. Furthermore, compared to the nitrate level in the seawater, the nitrate level at the surface of the sediment decreased dramatically to barely detectable level. This is the first record of nitrate exhaustion in the sediment–water interface of a cold seep. Given the dominance of Campylobacterota observed in the present and previous studies, this result suggested nitrate consumption by *Sulfurovum* and *Sulfurimonas* in the interface. Indeed, the *in situ* experiment showed nitrate expenditure occurring in the seawater around the seepage. Altogether, these results demonstrated that Campylobacterota played an important biogeochemical role in the nitrogen cycle of cold seeps by reducing nitrate to nitrogen. It is interesting that although CS47^T^ isolated from this study and a few other *Sulfurimonas* spp. possess nitrogen fixation genes, the expression of these genes was not detected, suggesting that *Sulfurimonas* did not perform nitrogen fixation in the cold seep. In contrast, nitrogen fixation genes belonging to ANME and methanogens, which are known to be capable of nitrogen fixation (38, 39), were expressed at high levels. Consistently, high concentrations of ammonia were detected in the RS. Based on these results, we propose that in the Formosa cold seep, Campylobacterota reduces nitrate to nitrogen, which is then transformed to ammonium by ANME and methanogens.

In this study, high concentrations of sulfide were detected in the top surface of RS, suggesting the existence of active anaerobic oxidation of methane in this region that led to the production of H_2_S. The H_2_S thus generated would diffuse into the surrounding water and reach the nearby macrobenthic area. Indeed, a previous study of the Formosa cold seep showed that the H_2_S concentration was up to 20 µM in the seawater of the animal community but could not be detected farther away from the seepage (23). The abundance of *Sulfurovum* closely correlated with the H_2_S level and was very high in the seawater in the animal community and very low or undetectable in the seawater farther away from the seepage (23). These observations indicated that H_2_S was an important factor affecting the distribution of Campylobacterota in the seawater. In the RS, Campylobacterota was mainly distributed in the upper areas, mostly the top surface. It is of note that the abundance/scarcity pattern of Campylobacterota appeared to be similar to that of nitrate, which had a high concentration in the seawater close to the top surface but dropped sharply in the subsurface and deeper regions of the sediments. These results suggested that nitrate was likely a key factor in determining the distribution of Campylobacterota in the RS.

**Fig 6:**
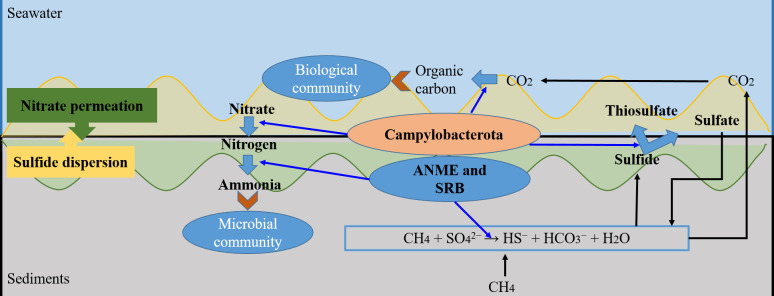
A sketch of proposed nitrogen, sulfur, and carbon cycling driven by Campylobacterota in the sediment–water interface of Formosa cold seep. The anaerobic methane-oxidizing archaea (ANME) and sulfate-reducing bacteria (SRB) perform AOM in the upper sediments, producing sulfide, which diffuses into the water and supports Campylobacterota as an electron donor, where the sulfide is completely or partially oxidized to form thiosulfate and sulfate. At the same time, nitrate at the sediment–water interface is consumed by Campylobacterota as an electron acceptor and is transformed into nitrogen, which is then fixed by ANME and methanogens to form ammonium, providing a nitrogen source for the local microbial community. Additionally, the carbon dioxide generated by the AOM reaction can be fixed by Campylobacterota to form organic matter, which serves as nutrients for the organisms in the cold seep.

In conclusion, in this study, we systematically analyzed the *in situ* activity and function of Campylobacterota in the Formosa cold seep. We found that Campylobacterota plays an important geochemical role in the sediment–water interface as illustrated in Fig. 6. The ANME and sulfate-reducing bacteria (SRB) probably oxidize anaerobically the methane in the upper RS, leading to the production of a large amount of sulfide. The sulfide then diffuses into the water and is utilized by Campylobacterota as an electron donor, producing thiosulfate and sulfate during the process. At the same time, nitrate as an electron donor is consumed heavily by Campylobacterota at the sediment–water interface, resulting in a sharp decline of the nitrate concentration in the upper sediments. Campylobacterota transforms nitrate into nitrogen, which is then fixed by ANME and methanogen to form ammonium, providing a sufficient nitrogen source for the local microbial community. Additionally, the carbon dioxide generated by the anaerobic oxidation of methane (AOM) can be used by Campylobacterota to form organic matter, providing nutrients for organisms in the cold seep.

### Description of *Sulfurovum fonticola sp. nov*

*Sulfurovum fonticola* (fon.ti'co.la. L. masc. *n*. *fons*, *fontis* spring, source; L. suff. *-cola* [from L. masc. or fem. *n*. *incola*] inhabitant, dweller; *N*.L. masc. *n*. fonticola an inhabitant of a source or a seep).

Cells are Gram-stain-negative, non-motile, ellipsoidal to short rod-shaped, 1.5–3.0 µm long, and 0.8–1.0 µm wide. Obligate anaerobic. Growth occurs at 5°C–20°C (optimum 10°C), pH 5.5–8.5 (optimum 6.5), and 2%–4% (wt/vol) NaCl (optimum 3% [wt/vol]). Obligate chemolithoautotrophic growth occurs with H_2_ as electron the sole donor and nitrate as the sole electron acceptor. Organic substrates are not utilized as carbon sources.

The type strain, CS14^T^ (=CGMCC 1.18034^T^ =MCCC 1K08662^T^) was isolated from the deep-sea cold seep in the SCS. The genomic DNA G + C content is 37.6 mol%.

### Description of *Sulfurimonas fonticola* sp. nov

*Sulfurimonas fonticola* (fon.ti'co.la. L. masc. *n*. *fons*, *fontis* spring, source; L. suff. *-cola* [from L. masc. or fem. *n*. *incola*] inhabitant, dweller; *N*.L. masc. *n*. fonticola an inhabitant of a source or a seep).

Cells are Gram-stain-negative, curved, 1.5–2.5 µm long, and 0.3–0.5 µm wide, motile by a polar flagellum. Anaerobic to microaerobic. Grows occurs at 0°C–37°C (optimum 25°C–28°C), pH 6.5–7.5 (optimum 6.5), and 1%–6% (wt/vol) NaCl (optimum 2%–3% [wt/vol]). Obligate chemolithoautotrophic growth occurs with H_2_ or S^0^ as electron donors and nitrate or molecular oxygen as electron acceptor. Organic substrates are not utilized as carbon sources.

The type strain, CS47^T^ (=CGMCC 1.18035^T^ =MCCC 1K08663^T^) was isolated from the deep-sea cold seep in the SCS. The genomic DNA G + C content is 32.1 mol%.

## Data Availability

The amplicon sequencing data were deposited in the Sequence Read Archive (National Center for Biotechnology Information) under the accession number PRJNA917276. The 16S rRNA gene sequences of strain CS14^T^ and CS47^T^ were deposited in GenBank under the accession numbers OQ152601 and OQ152602, respectively. The complete genome sequences of strain CS14^T^ and CS47^T^ were deposited in GenBank under the accession numbers PRJNA917258 and PRJNA917262 , respectively. Metatranscriptomic sequences were deposited in the Sequence Read Archive under the accession number PRJNA917539.

## SUPPLEMENTAL MATERIAL

Figure S1 mbio.00117-23-s0001.docx Transmission electron micrographs.

Figure S2 mbio.00117-23-s0002.docx Phylogenomic tree.

Table S1 mbio.00117-23-s0003.docx The genomes of *Sulfurovum* and *Sulfurimonas* used in this study.

Table S2 mbio.00117-23-s0004.docx Cellular fatty acid compositions of CS14T and CS47T and other members of *Sulfurovum* and *Sulfurimonas*.

Table S3 mbio.00117-23-s0005.docx The ANI and DDH values of strain CS14T and strain CS47T with other members of *Sulfurovum* and *Sulfurimonas*.

Table S4 mbio.00117-23-s0006.docx Comparison of the key sulfur and nitrogen metabolisms in *Sulfurovum* and *Sulfurimoans*.

Table S5 mbio.00117-23-s0007.docx Expression of the genes associated with energy metabolisms and carbon fixation in *Sulfurovum*.

Table S6 mbio.00117-23-s0008.docx Expression of the genes associated with energy metabolisms in *Sulfurimonas*.

Table S7 mbio.00117-23-s0009.docx Expression of nitrogenase in microbial communities.

ASM does not own the copyrights to Supplemental Material that may be linked to, or accessed through, an article. The authors have granted ASM a non-exclusive, world-wide license to publish the Supplemental Material files. Please contact the corresponding author directly for reuse.
