# Supplementary figures and images for: Nitrogen and sulfur cycling driven by Campylobacterota in the sediment–water interface of deep-sea cold seep: a case in the South China Sea
Source: mBio. 2023 Jul 6;14(4):e00117-23. doi: 10.1128/mbio.00117-23 (PMC10470523; doi:10.1128/mbio.00117-23)

**Figure S1.** Transmission electron micrographs of the cells of strains CS14T (A)and CS47T (B).


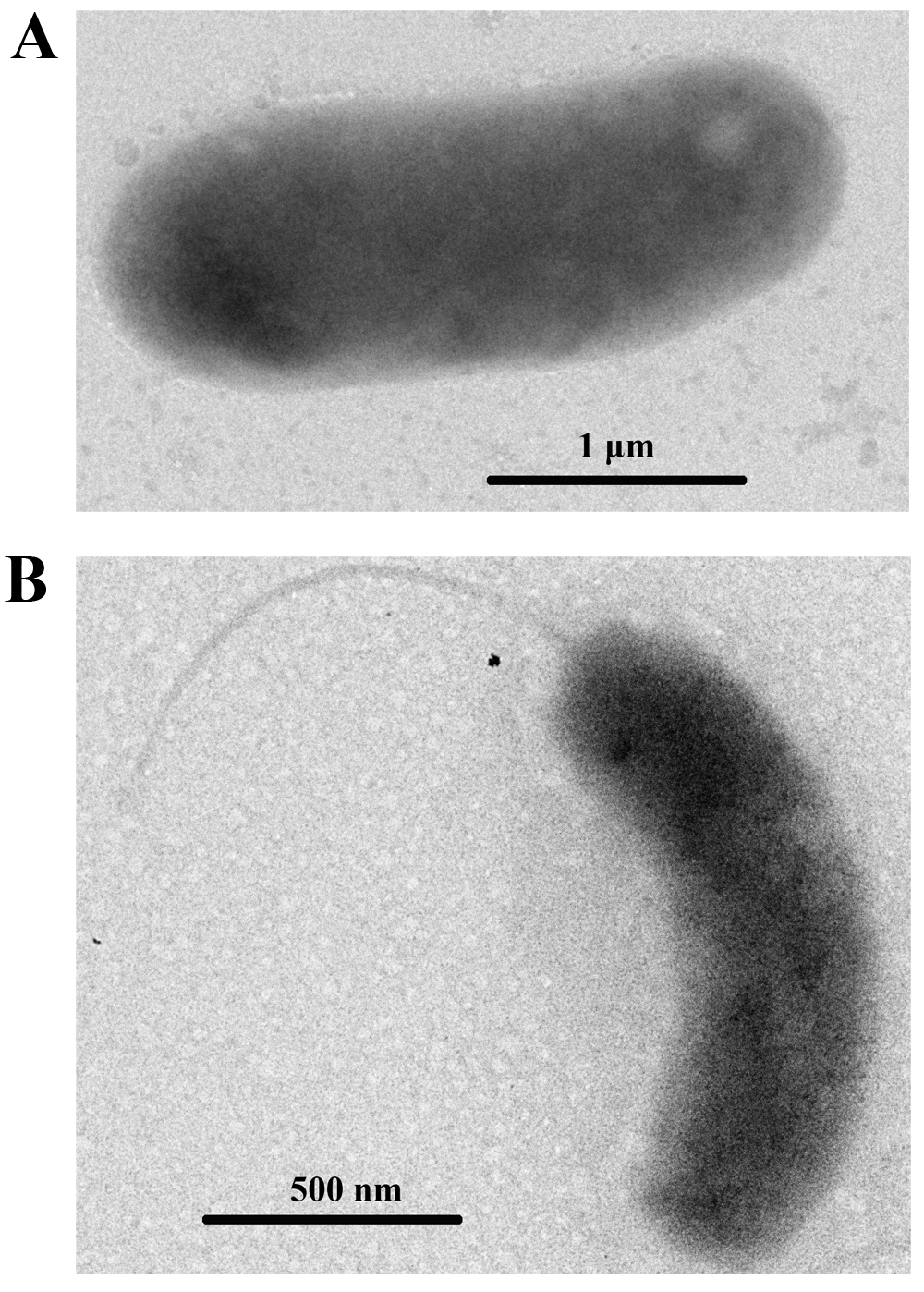

Supplement: Figure S1 — Transmission electron micrographs. [file mbio.00117-23-s0001.docx]
