# Supplementary material for: Nitrogen and sulfur cycling driven by Campylobacterota in the sediment–water interface of deep-sea cold seep: a case in the South China Sea
Source: mBio. 2023 Jul 6;14(4):e00117-23. doi: 10.1128/mbio.00117-23 (PMC10470523; doi:10.1128/mbio.00117-23)

**Figure S2.** Phylogenomic tree showing the positions of strains CS14^T^ and CS47^T^ and closely related taxa in *Sulfurovum* and *Sulfurimonas* using the maximum-likelihood algorithm. Bar, 0.1 substitution per position.


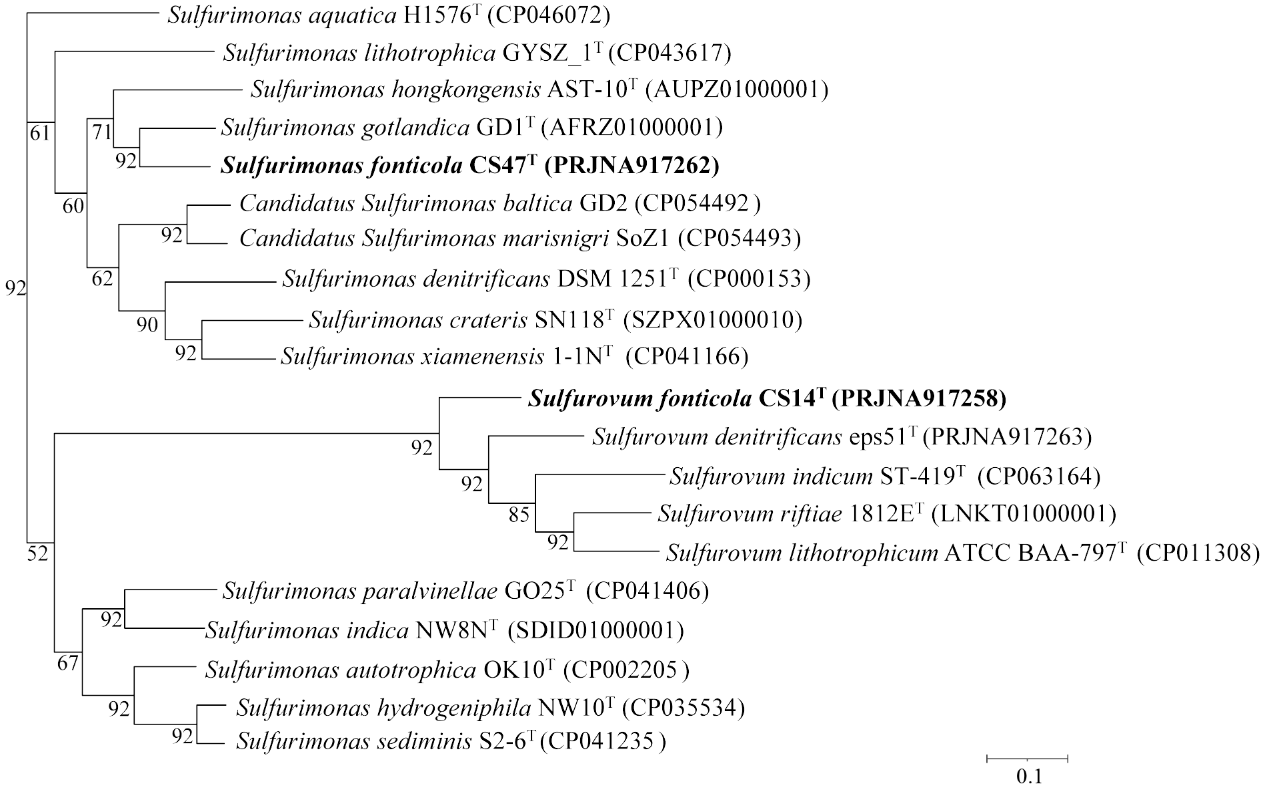

Supplement: Figure S2 — Phylogenomic tree. [file mbio.00117-23-s0002.docx]
