# Supplementary material for: Nitrogen and sulfur cycling driven by Campylobacterota in the sediment–water interface of deep-sea cold seep: a case in the South China Sea
Source: mBio. 2023 Jul 6;14(4):e00117-23. doi: 10.1128/mbio.00117-23 (PMC10470523; doi:10.1128/mbio.00117-23)
Supplement: Table S1 — The genomes of Sulfurovum and Sulfurimonas used in this study. [file mbio.00117-23-s0003.docx]

**Table S1.** The genomes of *Sulfurovum* and *Sulfurimonas* used in this study.

| **Organism** | **GenBank accession no.** |
| --- | --- |
| *Sulfurovum fonticola* CS14^T^ | PRJNA917258 |
| *Sulfurimonas fonticola* CS47^T^ | PRJNA917262 |
| *Sulfurovum denitrificans* DSM 19611^T^ | PRJNA917263 |
| *Sulfurovum indicum* ST-419^T^ | GCA_014931715.1 |
| *Sulfurovum riftiae* 1812E^T^ | GCA_001595645.1 |
| *Sulfurovum lithotrophicum* ATCC BAA-797^T^ | GCA_000987835.1 |
| *Sulfurimonas aquatic* H1576^T^ | GCA_017357825.1 |
| *Sulfurimonas autotrophica* DSM 16294^T^ | GCA_000147355.1 |
| *Sulfurimonas denitrificans* DSM 1251^T^ | GCA_000012965.1 |
| *Sulfurimonas gotlandica* GD1^T^ | GCA_000242915.2 |
| *Sulfurimonas lithotrophica* GYSZ_1^T^ | GCA_009258225.1 |
| *Sulfurimonas xiamenensis* 1-1N^T^ | GCA_009258045.1 |
| *Sulfurimonas hydrogeniphila* NW10^T^ | GCA_009068765.1 |
| *Sulfurimonas sediminis* S2-6^T^ | GCA_014905115.1 |
| *Sulfurimonas indica* NW8N^T^ | GCA_009192995.1 |
| Candidatus *Sulfurimonas marisnigri* SoZ1^T^ | GCA_015265475.1 |
| Candidatus *Sulfurimonas baltica* GD2^T^ | GCA_015265455.1 |
| Sulfurimonas *paralvinellae* GO25^T^ | GCA_014905135.1 |
| Sulfurimonas *crateris* SN118^T^ | GCA_005217605.1 |
| Sulfurimonas *hongkongensis* AST-10^T^ | GCA_000445475.1 |
| Sulfurimonas *marina* B2^T^ | GCA_014905095.1 |
