# Supplementary material for: Nitrogen and sulfur cycling driven by Campylobacterota in the sediment–water interface of deep-sea cold seep: a case in the South China Sea
Source: mBio. 2023 Jul 6;14(4):e00117-23. doi: 10.1128/mbio.00117-23 (PMC10470523; doi:10.1128/mbio.00117-23)
Supplement: Table S2 — Cellular fatty acid compositions of CS14T and CS47T and other members of Sulfurovum and Sulfurimonas. [file mbio.00117-23-s0004.docx]

**Table S2.** Cellular fatty acid compositions of CS14^T^ and CS47^T^ and other members of *Sulfurovum* and *Sulfurimonas.* 1, *Sulfurovum fonticola* CS14^T^; 2, *Sulfurovum denitrificans* DSM 19611^T^; 3, *Sulfurovum lithotrophicum* JCM 12117^T^; 4, *Sulfurovum riftiae* JCM 30810^T^; 5, *Sulfurimonas fonticola* CS47^T^; 6, *Sulfurimonas gotlandica* JCM 16533^T^; 7, *Sulfurimonas autotrophica* JCM 11897^T^. All data are from this study. Fatty acids that represent < 0.5% in all columns are omitted. Fatty acids that represent > 5.0% are in bold. -, Not detected.

| Fatty acid | 1 | 2 | 3 | 4 | 5 | 6 | 7 |
| --- | --- | --- | --- | --- | --- | --- | --- |
| **Saturated** |  |  |  |  |  |  |  |
| C_12:0_ | **9.2** | 0.2 | 2.4 | 0.5 | 0.1 | 0.1 | 1.3 |
| C_14:0_ | **12.3** | **8.9** | 2.2 | **7.6** | **8.1** | 3.0 | 4.9 |
| C_16:0_ | **12.7** | **18.9** | **29.1** | **21.3** | **24.5** | **16.9** | **30.4** |
| C_18:0_ | - | - | 0.8 | 0.5 | 0.1 | - | 1.2 |
| **Unsaturated** |  |  |  |  |  |  |  |
| C_16:1_ *ω*5c | 1.5 | 1.7 | 0.7 | 1.9 | 0.6 | 1.0 | 0.5 |
| C_16:1_ *ω*7c | **48.8** | **52.8** | **46.2** | **47.0** | **49.4** | **66.1** | **41.5** |
| **Summed features:†** |  |  |  |  |  |  |  |
| 2(C_12:0_ 3-OH; C_13:0_ DMA) | 0.2 | - | 2.9 | 0.5 | 0 | - | - |
| 5(C_15:0_ DMA; C_14:0_ 3-OH) | **5.5** | 3.2 | 0.4 | 3.0 | **7.8** | **7.0** | **7.0** |
| 10(C_18:1_ *ω*7*c*/*ω*9*t*/*ω12t*; Unknown) | **8.9** | **14.2** | **15.2** | **17.3** | **8.9** | **5.1** | **11.7** |
