# Supplementary material for: Nitrogen and sulfur cycling driven by Campylobacterota in the sediment–water interface of deep-sea cold seep: a case in the South China Sea
Source: mBio. 2023 Jul 6;14(4):e00117-23. doi: 10.1128/mbio.00117-23 (PMC10470523; doi:10.1128/mbio.00117-23)
Supplement: Table S3 — The ANI and DDH values of strain CS14T and strain CS47T with other members of Sulfurovum and Sulfurimonas. [file mbio.00117-23-s0005.docx]

**Table S3.** The ANI and DDH values of strain CS14^T^ and strain CS47^T^ with other members of *Sulfurovum* and *Sulfurimonas.*

| ANI/DDH value | CS14^T^ |
| --- | --- |
| *Sulfurovum denitrificans* DSM 19611^T^ | 75.05%/21.20% |
| *Sulfurovum riftiae* 1812E^T^ | 73.04%/20.00% |
| *Sulfurovum lithotrophicum* 42BKT^T^ | 72.96%/19.70% |
| *Sulfurovum indicum* ST-419^T^ | 72.76%/19.70% |
| ANI/DDH value | CS47^T^ |
| *Sulfurimonas gotlandica* GD1^T^ | 81.38%/24.20% |
| *Sulfurimonas hongkongensis* AST-10^T^ | 76.91%/20.80% |
| Candidatus *Sulfurimonas marisnigri* SoZ1 | 75.66%/20.70% |
| Candidatus *Sulfurimonas baltica* GD2 | 75.39%/20.60% |
| *Sulfurimonas xiamenensis* 1-1N^T^ | 74.57%/20.50% |
| *Sulfurimonas denitrificans* DSM 1251^T^ | 73.83%/19.80% |
| *Sulfurimonas aquatica* H1576^T^ | 73.58%/20.20% |
| *Sulfurimonas lithotrophica* GYSZ_1^T^ | 73.45%/19.40% |
| *Sulfurimonas crateris* SN118^T^ | 73.05%/18.70% |
| *Sulfurimonas autotrophica* OK10^T^ | 72.79%/19.30% |
| *Sulfurimonas indica* NW8N^T^ | 72.38%/19.10% |
| *Sulfurimonas sediminis* S2-6^T^ | 72.18%/18.90% |
| *Sulfurimonas hydrogeniphila* NW10^T^ | 72.15%/19.10% |
| *Sulfurimonas marina* B2^T^ | 71.97%/19.20% |
| *Sulfurimonas paralvinellae* GO25^T^ | 71.68%/19.00% |
