# Supplementary material for: Nitrogen and sulfur cycling driven by Campylobacterota in the sediment–water interface of deep-sea cold seep: a case in the South China Sea
Source: mBio. 2023 Jul 6;14(4):e00117-23. doi: 10.1128/mbio.00117-23 (PMC10470523; doi:10.1128/mbio.00117-23)
Supplement: Table S4 — Comparison of the key sulfur and nitrogen metabolisms in Sulfurovum and Sulfurimoans. [file mbio.00117-23-s0006.docx]

**Table S4.** Comparison of the key sulfur and nitrogen metabolisms in *Sulfurovum* and *Sulfurimoans*. 1, *Sulfurovum fonticola*; 2, *Sulfurovum denitrificans*; 3, *Sulfurovum lithotrophicum*; 4, *Sulfurovum riftiae*; 5, *Sulfurovum indicum*; 6, *Sulfurimonas fonticola*; 7, *Sulfurimonas gotlandica*; 8, *Sulfurimonas autotrophica*; 9, *Sulfurimonas aquatica*; 10, *Sulfurimonas sediminis*; 11, *Sulfurimonas hydrogeniphila*; 12, Candidatus *Sulfurimonas marisnigri*; 13, Candidatus *Sulfurimonas baltica*; 14, *Sulfurimonas indica*; 15, *Sulfurimonas xiamenensis*; 16, *Sulfurimonas lithotrophica*; 17, *Sulfurimonas crateris*; 18, *Sulfurimonas hongkongensis*; 19, *Sulfurimonas paralvinellae*; 20, *Sulfurimonas denitrificans*; 21, *Sulfurimonas marina*.

|  | *soxCDYZ* | *soxABXYZ* | *sqr* | [Denitrification](javascript:;) | Assimilation nitrate reduction | Dissimilation  nitrate reduction | [Nitrogen fixation](javascript:;) |
| --- | --- | --- | --- | --- | --- | --- | --- |
| 1 | + | - | +(4) | + | - | - | - |
| 2 | + | + | +(6) | + | - | - | - |
| 3 | + | + | +(3) | + | - | - | - |
| 4 | + | + | +(5) | + | - | - | - |
| 5 | + | + | +(5) | + | - | - | - |
| 6 | + | - | +(5) | + | + | - | + |
| 7 | + | + | +(5) | + | - | - | - |
| 8 | + | + | +(5) | + | - | - | - |
| 9 | + | + | +(3) | - | + | - | - |
| 10 | + | + | +(2) | + | - | - | - |
| 11 | + | + | +(4) | + | - | - | - |
| 12 | + | + | +(4) | - | + | - | + |
| 13 | + | + | +(4) | - | + | + | + |
| 14 | + | + | +(3) | + | - | - | - |
| 15 | + | - | +(3) | + | - | - | - |
| 16 | + | - | +(3) | + | + | - | + |
| 17 | + | + | +(2) | + | - | - | - |
| 18 | + | + | +(5) | + | - | - | - |
| 19 | + | + | +(3) | + | - | - | - |
| 20 | + | + | +(3) | + | - | - | - |
| 21 | + | + | +(4) | - | - | - | + |
