# Supplementary material for: Nitrogen and sulfur cycling driven by Campylobacterota in the sediment–water interface of deep-sea cold seep: a case in the South China Sea
Source: mBio. 2023 Jul 6;14(4):e00117-23. doi: 10.1128/mbio.00117-23 (PMC10470523; doi:10.1128/mbio.00117-23)
Supplement: Table S5 — Expression of the genes associated with energy metabolisms and carbon fixation in Sulfurovum. [file mbio.00117-23-s0007.docx]

**Table S5.** Expression of the genes associated with energy metabolisms and carbon fixation in *Sulfurovum*. Fragments per kilobase of transcript per million fragments mapped (FPKM) are used to describe the expression level, and the top one is shown.

| Gene | SC-1 | SC-2 | SC-3 | RS |
| --- | --- | --- | --- | --- |
| **Hydrogen oxidation** | | |  |  |
| *hydB* | 0 | 0 | 0 | 111.6 |
| **Sulfide oxidation** | | | | |
| *sqr* | 15.0 | 411.8 | 4061.6 | 302.9 |
| **Sulfur oxidation** | | | | |
| *soxA* | 2.9 | 34.3 | 31.1 | 0 |
| *soxB* | 12.7 | 265.0 | 667.7 | 0 |
| *soxC* | 26.2 | 169.4 | 164.7 | 63.7 |
| *soxD* | 21.8 | 114.6 | 239.6 | 0 |
| *soxX* | 2.4 | 67.6 | 1390.2 | 0 |
| *soxY* | 38.2 | 177.3 | 70.5 | 0 |
| *soxZ* | 5.8 | 68.8 | 17.5 | 0 |
| **Denitrification** | | | | |
| *napA* | 225.0 | 1076.6 | 3130.6 | 53.6 |
| *napB* | 24.9 | 276.8 | 402.1 | 105.3 |
| *nirS* | 1.0 | 16.5 | 3685.2 | 46.0 |
| *norB* | 13.0 | 3.5 | 140.1 | 111.3 |
| *norC* | 5.6 | 3.9 | 115.5 | 0 |
| *nosZ* | 0.4 | 12.4 | 1425.4 | 117.6 |
| **Carbon fixation** | | | | |
| *ATP-dependent citrate lyase* | 42.2 | 155.9 | 633.9 | 181.7 |
