# Supplementary material for: Nitrogen and sulfur cycling driven by Campylobacterota in the sediment–water interface of deep-sea cold seep: a case in the South China Sea
Source: mBio. 2023 Jul 6;14(4):e00117-23. doi: 10.1128/mbio.00117-23 (PMC10470523; doi:10.1128/mbio.00117-23)
Supplement: Table S6 — Expression of the genes associated with energy metabolisms in Sulfurimonas. [file mbio.00117-23-s0008.docx]

**Table S6.** Expression of the genes associated with energy metabolisms in *Sulfurimonas*. Fragments per kilobase of transcript per million fragments mapped (FPKM) are used to describe the expression level and the top one is shown.

| Gene | SC-1 | SC-2 | SC-3 | RS |
| --- | --- | --- | --- | --- |
| **Hydrogen oxidation** |  |  |  |  |
| *hydB* | 0 | 0 | 0 | 153.5 |
| **Sulfide oxidation** |  |  |  |  |
| *sqr* | 0 | 0 | 0.39 | 214.6 |
| **Sulfur oxidation** |  |  |  |  |
| *soxB* | 0 | 0.6 | 0 | 0 |
| *soxC* | 2.3 | 214.9 | 3634.2 | 529.9 |
| *soxD* | 0.4 | 80.2 | 1265.7 | 0 |
| *soxX* | 0 | 0.47 | 0 | 0 |
| **Denitrification** |  |  |  |  |
| *napA* | 3.1 | 69.8 | 155.5 | 740.8 |
| *napB* | 0 | 0.6 | 0 | 234.8 |
| *nirS* | 0.2 | 0 | 7.3 | 167.3 |
| *norB* | 10.3 | 24.4 | 11.4 | 119.4 |
| *norC* | 0 | 0 | 0 | 27.1 |
