# Supplementary material for: Nitrogen and sulfur cycling driven by Campylobacterota in the sediment–water interface of deep-sea cold seep: a case in the South China Sea
Source: mBio. 2023 Jul 6;14(4):e00117-23. doi: 10.1128/mbio.00117-23 (PMC10470523; doi:10.1128/mbio.00117-23)
Supplement: Table S7 — Expression of nitrogenase in microbial communities. [file mbio.00117-23-s0009.docx]

**Table S7.** Expression of nitrogenase in microbial communities. Fragments per kilobase of transcript per million fragments mapped (FPKM) are used to describe the expression level.

| Gene | SC-1 | SC-2 | SC-3 | RS | Nr annotation |
| --- | --- | --- | --- | --- | --- |
| c164045_g1 | 0 | 0 | 0 | 272.26 | nitrogenase molybdenum-iron protein alpha chain [ANME-2 cluster archaeon] |
| c528227_g1 | 0 | 0 | 0 | 134.63 | nitrogenase molybdenum-iron protein alpha chain [*Methanolobus tindarius*] |
